# Do Pneumococcal Conjugate Vaccines Represent Good Value for Money in a Lower-Middle Income Country? A Cost-Utility Analysis in the Philippines

**DOI:** 10.1371/journal.pone.0131156

**Published:** 2015-07-01

**Authors:** Manuel Alexander Haasis, Joyce Anne Ceria, Wantanee Kulpeng, Yot Teerawattananon, Marissa Alejandria

**Affiliations:** 1 National Center for Pharmaceutical Access and Management (NCPAM), Department of Health, Manila, Philippines; 2 Health Intervention and Technology Assessment Program (HITAP), Department of Health, Ministry of Public Health, Nonthaburi, Thailand; 3 Institute of Clinical Epidemiology-National Institutes of Health (NIH), University of the Philippines, Manila, Philippines; Centers for Disease Control & Prevention, UNITED STATES

## Abstract

**Objectives:**

The objective of this study is to assess the value for money of introducing pneumococcal conjugate vaccines as part of the immunization program in a lower-middle income country, the Philippines, which is not eligible for GAVI support and lower vaccine prices. It also includes the newest clinical evidence evaluating the efficacy of PCV10, which is lacking in other previous studies.

**Methods:**

A cost-utility analysis was conducted. A Markov simulation model was constructed to examine the costs and consequences of PCV10 and PCV13 against the current scenario of no PCV vaccination for a lifetime horizon. A health system perspective was employed to explore different funding schemes, which include universal or partial vaccination coverage subsidized by the government. Results were presented as incremental cost-effectiveness ratios (ICERs) in Philippine peso (Php) per QALY gained (1 USD = 44.20 Php). Probabilistic sensitivity analysis was performed to determine the impact of parameter uncertainty.

**Results:**

With universal vaccination at a cost per dose of Php 624 for PCV10 and Php 700 for PCV13, both PCVs are cost-effective compared to no vaccination given the ceiling threshold of Php 120,000 per QALY gained, yielding ICERs of Php 68,182 and Php 54,510 for PCV10 and PCV13, respectively. Partial vaccination of 25% of the birth cohort resulted in significantly higher ICER values (Php 112,640 for PCV10 and Php 84,654 for PCV13) due to loss of herd protection. The budget impact analysis reveals that universal vaccination would cost Php 3.87 billion to 4.34 billion per annual, or 1.6 to 1.8 times the budget of the current national vaccination program.

**Conclusion:**

The inclusion of PCV in the national immunization program is recommended. PCV13 achieved better value for money compared to PCV10. However, the affordability and sustainability of PCV implementation over the long-term should be considered by decision makers.

## Introduction

*Streptococcus pneumoniae* (*S*. *pneumoniae*) can cause invasive pneumococcal diseases including bacterial meningitis, bacteremia and sepsis, as well as non-invasive pneumococcal diseases, such as pneumonia and acute otitis media (AOM). Pneumococcal conjugate vaccines (PCV) have been found safe and effective in preventing *S*. *pneumoniae*-related diseases in young children [1–3]. In addition to this direct vaccine effect, indirect vaccine effects, in particular herd protection, have also been documented. Clinical studies have shown that vaccinating infants and young children with PCV can reduce transmission of the bacterium and disease to unvaccinated populations [4, 5].

As a lower-middle income country, the Philippines is not eligible for GAVI support. Therefore, the Philippines faces substantial financial barriers to PCV implementation. As a result, the Philippine government is currently piloting PCV in only selected regions covering approximately 25%-30% of the total of 2 million eligible infants in the country [6]. In addition, the Philippine government remains undecided between the two vaccines available in the market and opted to pilot both the 10- and 13-valent vaccines in the national vaccination program.

A number of economic evaluations of PCV vaccination have been done worldwide. However, most studies used clinical outcomes derived from clinical studies of PCV7 and extrapolated clinical benefits for PCV10 and PCV13 [7–11]. This is because there was no randomized controlled trial (RCT) that directly assessed the benefit of PCV10 and PCV13. A recently published RCT on PCV10 versus hepatitis control vaccine demonstrates a higher vaccine efficacy against AOM compared to previous RCTs of PCVs (all seven-valent) [12, 13]. This timely economic evaluation aims to inform immunization implementation strategies regarding the likely impact, value for money and budget implications of PCV vaccination in the Philippines. The study focuses on whether to introduce a universal versus partial PCV vaccination program as well as on informing which vaccine type should represent the best value for money given the new information.

## Materials and Methods

### Markov Model

**Fig 1:**
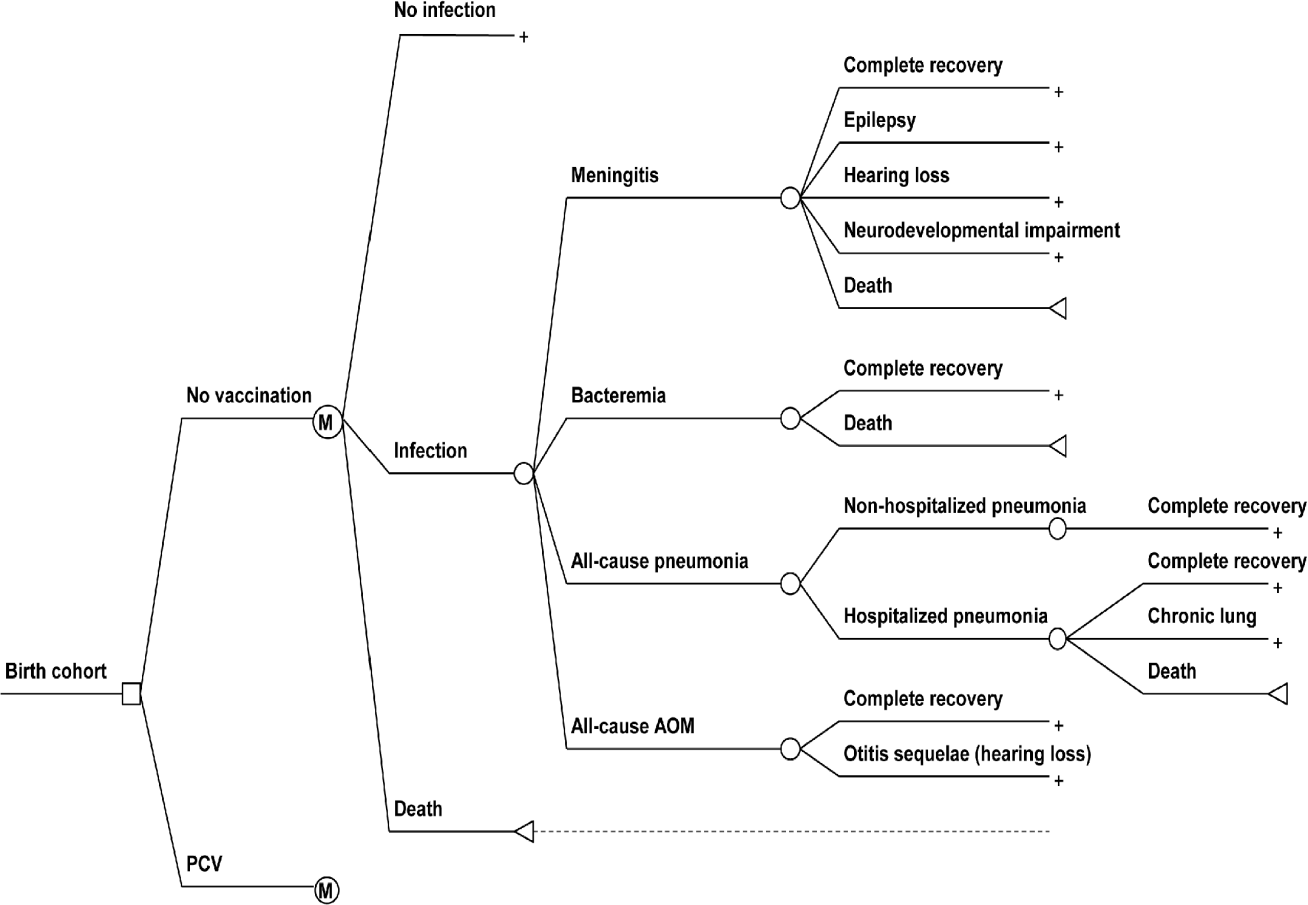
Markov model used for assessing the health and economic impact of pneumococcal conjugate vaccine (PCV) vaccination compared to ‘no vaccination’. The structure of the PCV node was omitted in the figure, as it is identical to the ‘no vaccination’ node.

A Markov model with one-year cycle length adapted from a prior study [14] was used to estimate the lifetime costs and outcomes for PCV10 and PCV13 compared to ‘no vaccination’, consisting of three major health states: good health, *S*. *pneumoniae* infection and death (Fig 1). Whilst the model structure for this study was adapted from Thai setting, this model has been validated in consultation meetings by Philippine experts including infectious disease specialists, epidemiologists, health economists, and policy decision makers.

It was assumed that only one infection per cycle could occur. The analysis adopted a health system perspective, which includes direct costs of PCV related treatments and of the vaccination program. An annual discount rate of 3.5% for both costs and outcomes was applied based on recommendations by the Formulary Executive Council (FEC), which is responsible for the development of the Philippine National Formulary. All costs are presented for the year 2013.

### Intervention

The effects of switching from no vaccination to either PCV10 or PCV13 were evaluated, alternatively with and without indirect effects. For this study, a 2-dose primary series at 1.5 and 2.5 months, plus a booster dose at 9 months of age (2+1) was considered. Vaccine coverage rates of 90%, 88% and 86% for the first, second and booster dose, respectively, were assumed to correspond the achieved 2013 coverage rates for DPT-HepB-Hib vaccination for the first two doses and for measles vaccination administered at the same time as the booster dose [15].

### Policy scenarios

The study employs two potential policy scenarios, which examine the impact of different vaccination coverage rates on health outcomes, cost-effectiveness and government budget.

*Scenario 1* *(Universal coverage)*: 100% of the country’s birth cohort will receive PCV vaccination fully funded by the government (vaccination is free of charge to the vaccinee).

*Scenario 2* *(Partial coverage*, *status quo)*: 25% of the country’s birth cohort will receive PCV vaccination fully funded by the government. The remaining 75% of the birth cohort will not be vaccinated.

### Model input parameters

#### Epidemiological parameters

A summary of the epidemiological parameters used in the analysis is provided in Table 1. Philippine-specific data on the incidence of pneumococcal meningitis, bacteremia and sepsis in children <5 years were taken from the PneumoNet study [16]. No published data were available on the IPD incidence among Filipinos older than 5 years of age. Therefore, incidences of pneumococcal meningitis and bacteremia/sepsis in the >5 age groups were derived based on Thai data [17–19] and were adjusted according to the following formula:

$$\left(1\right)\;\text{PHL pn. meningitis incidence for age groups}\geq 5=\text{Thai pn. meningitis incidence}\;\times \;\frac{\text{PHL pn. meningitis incidence}<5}{\text{Thai pn. meningitis incidence}<5}$$

$$\left(2\right)\;\text{PHL pn. bacteremia incidence for age groups}\geq 5=\text{Thai pn. bacteremia incidence}\;\times \;\frac{\text{PHL pn. bacteremia incidence}<5}{\text{Thai pn. bacteremia incidence}<5}$$

**Table 1:** Input parameters used in the model.

Parameter description			Distribution	Mean	SE	References
**Epidemiological parameters**	Pneumococcal (pn) meningitis in age:					
		0–4	Beta	0.00011	0.000026	[16]
		5–9	Beta	0.00002	0.000002	[14, 16]
		10–14	Beta	0.00003	0.000003	[14, 16]
		15–24	Beta	0.00002	0.000002	[14, 16]
		25–34	Beta	0.00003	0.000002	[14, 16]
		35–44	Beta	0.00003	0.000002	[14, 16]
		45–54	Beta	0.00003	0.000002	[14, 16]
		55–64	Beta	0.00003	0.000002	[14, 16]
		65 and above	Beta	0.00004	0.000003	[14, 16]
	Bacteremia and sepsis in age:					
		0–4	Beta	0.0000568	0.000019	[16]
		5–19	Beta	0.0000191	0.000005	[16, 18]
		20–49	Beta	0.0000275	0.000004	[16, 18]
		50–64	Beta	0.0000506	0.000011	[16, 18]
		65 and above	Beta	0.0001434	0.000026	[16, 18]
	Hospitalized all-cause pneumonia in age:					
		0–5	Normal	0.02967	0.000426	[16]
		6–18	Normal	0.00173	0.000008	Philippine Health Insurance Corporation (PhilHealth)
		19–49	Normal	0.00084	0.000005	PhilHealth
		50 and above	Normal	0.00778	0.000024	PhilHealth
	AOM in age:					
		0–12	Normal	0.09570002	0.00005699	[20]
	Epilepsy after pn. meningitis		Beta	0.103448276	0.055601778	[21]
	Hearing loss after pn. Meningitis		Beta	0.034482759	0.03331351	[21]
	Neurodevelopmental impairment after pn. Meningitis		Beta	0.344827586	0.086779706	[21]
	Death after pn. meningitis		Beta	0.352941176	0.112638483	[16]
	Death after pn. bacteremia and sepsis		Beta	0.400	0.147709789	[16]
	Necrotizing pneumonia after pn. Pneumonia		Beta	0.183098592	0.04557861	[22]
	Death after hospitalized pneumonia		Beta	0.0215	0.01065	[23, 24]
	Hearing loss after AOM		Beta	0.050968271	0.000598046	[14]
	IPD with final diagnosis of meningitis		Beta	0.35	0.1126	[16]
	IPD with final diagnosis of sepsis		Beta	0.40	0.1477	[16]
**Cost parameters (Php)**	**Vaccine costs**					
	PCV 10 cost per dose		Not varied	624		DOH Expanded Program on Immunization (EPI)
	PCV 13 cost per dose		Not varied	700		DOH EPI
	**Direct medical cost**					
	Syringe		Not varied	2.50		DOH EPI
	Storage, warehousing and delivery		Not varied	8% of the vaccine price		DOH EPI
	Program cost		Not varied	1% of the vaccine price		
	**Cost per episode**					
	Pn meningitis in age:					
		0–5	Gamma	49560	24780	PhilHealth
		6–18	Gamma	47124	23562	PhilHealth
		19–49	Gamma	59373	29686.50	PhilHealth
		50 and above	Gamma	67751	33875.50	PhilHealth
	Pn bacteremia and sepsis in age:					
		0–5	Gamma	29754	14877	PhilHealth
		6–18	Gamma	44141	22070.50	PhilHealth
		19–49	Gamma	65622	32811	PhilHealth
		50 and above	Gamma	86762	43381	PhilHealth
	Hospitalized all-cause pneumonia in age:					
		0–5	Gamma	15284	7,642	PhilHealth
		6–18	Gamma	15809	7,905	PhilHealth
		19–49	Gamma	17661	8,831	PhilHealth
		50 and above	Gamma	21425	10,713	PhilHealth
	Non-hospitalized all-cause pneumonia in age:					
		0–5	Gamma	300	150	Expert panel
		6–18	Gamma	300	150	Expert panel
		19–49	Gamma	300	150	Expert panel
		50 and above	Gamma	300	150	Expert panel
	AOM in age:					
		0–5	Gamma	300	150	Expert panel
		6–18	Gamma	300	150	Expert panel
		19–49	Gamma	300	150	Expert panel
		50 and above	Gamma	300	150	Expert panel
	**Cost per year**					
	Epilepsy in age:					
		0–14	Gamma	6095.00	730.10	[14]
		15–59	Normal	2462.00	32.98	[14]
		60 and above	Gamma	2572.00	131.08	[14]
	Hearing loss in age:					
		0–14	Gamma	1379.00	593.04	[14]
		15–59	Gamma	1289.00	73.92	[14]
		60 and above	Gamma	2018.00	189.38	[14]
	Neurodevelopmental impairment in age:					
		0–14	Gamma	5511.00	3588.67	[14]
		15–59	Gamma	1440.00	110.23	[14]
		60 and above	Gamma	8940.00	4449.18	[14]
	Chronic lung in age:					
		0–14	Gamma	2161.00	2160.68	[14]
		15–59	Normal	5086.00	96.14	[14]
		60 and above	Normal	5594.00	48.35	[14]
**Utility parameters**	Meningitis		Beta	0.9638	0.0046	[14]
	Bacteremia and sepsis		Beta	0.9852	0.0025	[14]
	Pneumonia		Beta	0.9910	0.0020	[14]
	AOM		Beta	0.9984	0.0001	[14]
	Epilepsy		Beta	0.6400	0.0738	[14]
	Hearing loss		Beta	0.5500	0.0554	[14]
	Neurodevelopmental impairment					
	Mild mental retardation		Beta	0.6900	0.0707	[14]
	Severe mental retardation		Beta	0.1000	0.1085	[14]
	Mental retardation + epilepsy		Beta	0.0001	0.0943	[14]
	Chronic lung disease		Normal	0.5900	0.0575	[14]
	PCV7 Serotype coverage US in age:					
		20–39	Not varied	71.30%		[25]
		40–64	Not varied	65.40%		[25]
		65 and above	Not varied	69.70%		[25]
	% IPD fall among unvaccinated group in US in age:					
		20–39	Beta	40.00%	4.59%	[4]
		40–64	Beta	14.00%	4.59%	[4]
		65 and above	Beta	29.00%	3.57%	[4]

All meningitis and bacteremia/sepsis cases were assumed to have required hospitalization. Due to the lack of local information of sequelae, Thai data were used. Case-fatality ratios (CFR) for meningitis and bacteremia/sepsis were derived from the PneumoNet study [16].

All-cause hospitalized pneumonia incidences were gathered from the PneumoNet study and anonymized insurance claims were obtained from the Philippine Health Insurance Corporation (PhilHealth) [16]. Data on non-hospitalized pneumonia was unavailable; thus, information was obtained through an expert panel of infectious disease specialists who estimated a 60:40 ratio of hospitalized to non-hospitalized pneumonia cases in the Philippines. The probability of dying from hospitalized pneumonia was estimated as 2.15% [23, 24].

All-cause AOM incidence was derived from the 2012 national cross-sectional survey of 2,000 children in community health centers and schools in the Philippines [20]. It was assumed that none of the AOM cases were severe enough to require hospitalization or to cause death. According to global estimates, AOM occurs regularly in younger age groups [26]; thus, incidence of AOM was assumed to be zero for individuals that were 12 years or older.

**Fig 2:**
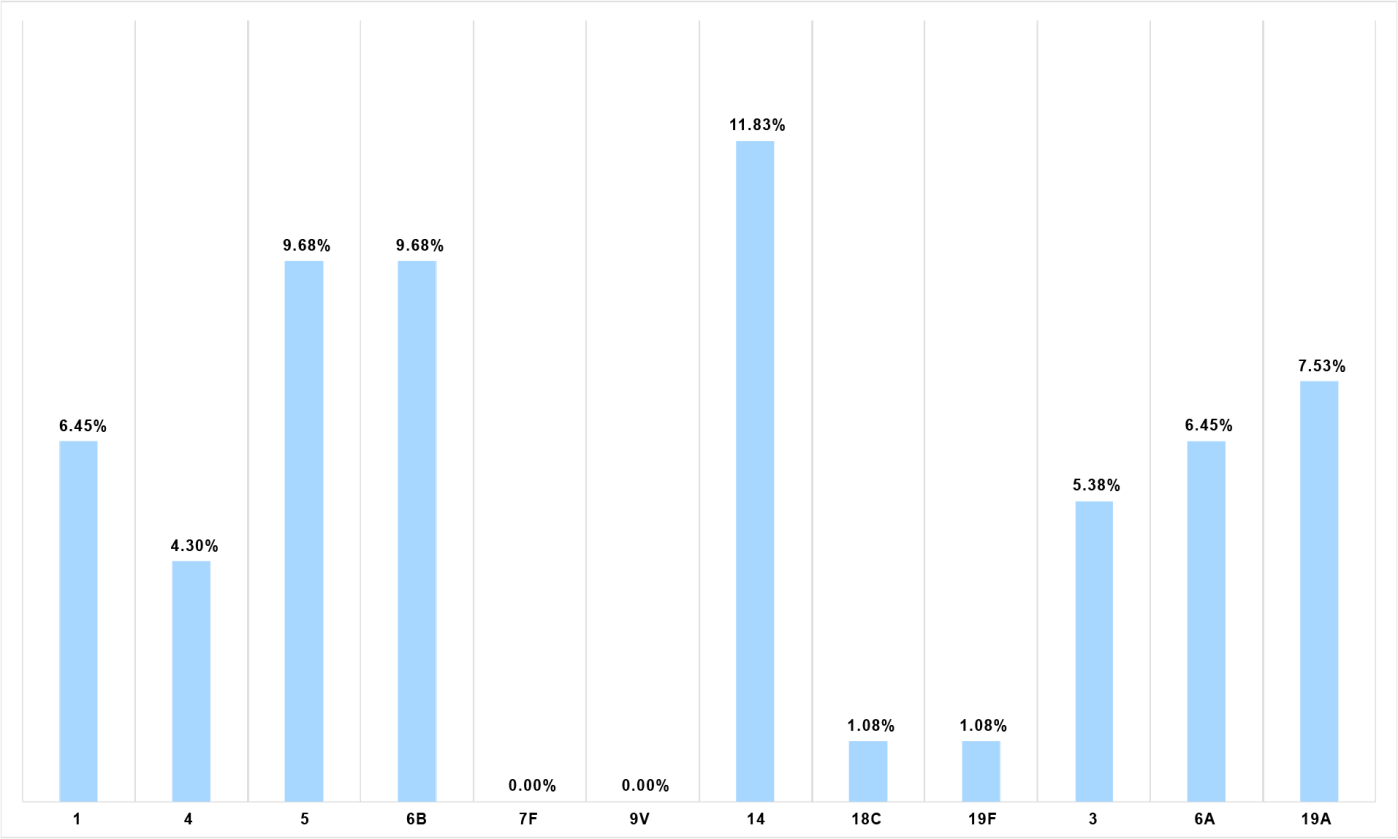
Proportion of IPD serotypes among Filipino children aged <5 years covered by PCV13 (N = 93).

The distribution of serotypes in invasive disease was taken from a comprehensive laboratory-based surveillance study involving 42 hospitals across the Philippines (Fig 2) [27]. The vaccine-type IPD coverage of PCV7, PCV10, and PCV13 was computed for different age groups (S1 Table).

#### Direct effects (vaccine efficacy)

Due to lacking evidence on vaccine efficacy (VE) for the 3+0 dosing schedule, efficacy of a 2-dose primary series plus a booster dose (2+1) was used. All efficacy estimates were based on the intention-to-treat (ITT) population.

For PCV13, demonstration of VE is based on non-inferior immunogenicity compared with PCV7 rather than efficacy trials measuring clinical endpoints [28, 29]. However, evidence supporting the effectiveness of PCV13 for covered serotypes has been documented in various settings [30–33]. VE against vaccine-type IPD for PCV13 for covered serotypes was assumed to be the same (92%) as for PCV10, which was obtained from the Finnish Invasive Pneumococcal Disease (FinIP) vaccine trial with a 2+1 dosing schedule [34]. The overall PCV10 efficacy against IPD (46.49%) was calculated by multiplying PCV10 vaccine-type efficacy against IPD (92%) by the local serotype coverage of PCV10 (50.54%). The overall PCV13 efficacy against IPD (64.30%) was derived by multiplying PCV10 vaccine-type efficacy against IPD (92%) by the local serotype coverage of PCV13 (69.89%) (Table 2).

**Table 2:** Vaccine efficacy by syndrome adjusted by 2+1 schedule and local serotype distribution.

Health conditions	Adjusted overall VE using Philippine serotype coverage	
	PCV10	PCV13
**IPD all serotypes**	46.49%^a^	64.30%^b^
**Clinical pneumonia**	8.42%^c^	11.64%^d^
**Clinical AOM**	18.38%^e^	25.43%^f^

a- *PCV*10_2+1_ *VE against vaccine type IPD* × *local vaccine serotype IPD coverage of PCV*10

b- *PCV*10_2+1_ *VE against vaccine type IPD* × *local vaccine serotype IPD coverage of PCV*13

c- *PCV*10_3+1_ *VE against clinical pneumonia* × (1−0.0324)

d- *PCV*10_3+1_ *VE against clinical pneumonia* × (1−0.0324) × (*local vaccine serotype IPD coverage of PCV*13/*local vaccine serotype IPD coverage of PCV*10)

e- *PCV*10_3+1_ *VE against clinical AOM* × (1−0.0324)

f- *PCV*10_3+1_ *VE against clinical AOM* × (1−0.0324) × (*local vaccine serotype IPD coverage of PCV*13/*local vaccine serotype IPD coverage of PCV*10)

VE of a 2+1 dosing schedule of PCV10 and PCV13 against all-cause pneumonia and AOM was calculated based on the results of the recently published double-blind randomized controlled ‘Clinical Otitis Media and Pneumonia Study’ (COMPAS) [13], which examines the efficacy of PCV10 with a 3+1 schedule. Compared to PCV10, PCV13 efficacies against all-cause pneumonia and AOM were assumed to increase proportionally with the increase in vaccine serotype IPD coverage (Table 2).

This study did not assume efficacy against AOM caused by nontypeable *Haemophilus influenzae* (NTHi) based on recent findings in the COMPAS trial where the results were not statistically significant (21.5%; 95% CI: -43.4–57.0) [13]. Also, regulatory authorities in the Philippines and Europe do not recognize the claimed protective effect of PCV10 against NTHi [35, 36].

Compared to the 3+1 schedule, VE for the 2+1 schedule was adjusted to take into account reduced immunogenicity for serotypes 6B and 23F based on a 2011 systematic review and meta-analysis [37]. The reduction in VE was assumed to be 20% for each of these serotypes. In Filipino children below 5 years of age, serotypes 6B and 23F comprised 16.2% of invasive pneumococcal isolates covered in PCV7, reducing overall VE for the 2+1 schedule by 3.24% (16.2% of 20% reduction in vaccine efficacy) compared to the 3+1 schedule. The following formula was used to adjust vaccine efficacy derived from a 3+1 dosing schedule to a 2+1 schedule:

$$VE_{2+1}=VE_{3+1}\;\times \;\left(1-0.0324\right)$$

#### Indirect effects (herd protection)

Besides estimating the direct effects of the vaccine, the model also assessed the vaccine’s indirect effects against IPD among the unvaccinated population. Herd protection was assumed to be realized at a vaccination coverage rate of 80% [38, 39]. The percentage change in IPD infections among unvaccinated individuals was based on the percentage decline of IPD incidence in the United States following the introduction of routine vaccination of PCV7 for infants and young children [4], adjusted for the difference between the USA and the Philippine serotype coverage. Expected IPD percentage reduction for the Philippines and the USA is reflected for different age groups in S2 Table.

This study considered herd protection for pneumococcal meningitis, bacteremia/sepsis, and pneumonia. In order to estimate the percentage change for pneumonia among unvaccinated populations, it was assumed that pneumonia incidence decreases proportionally to the IPD fall for respective age groups, adjusted by the ratio of pneumococcal pneumonia to hospitalized pneumonia cases.

Duration of vaccine protection was assumed to be 5 years for both direct and indirect effects, which is in line with other PCV economic evaluations [40–42].

#### Vaccine costs

Costs were converted into Philippine peso using the mean exchange rate between the US Dollar and the Philippine peso (Php) in December 2013 (1 USD = 44.20 Php) [43]. In 2013, the Philippine government was offered to procure PCV10 and PCV13 for Php 624 and Php 700 per dose, respectively, based on 2013 reference prices of the Philippine EPI, while the market price is between Php 2,261 to Php 3,670 [44]. Additional costs for syringe, storage, warehousing, delivery and program implementation were considered (Table 1). Taxes, handling fee and freight cost incurred through UNICEF procurement were excluded since the national program is considering local bidding as its mode of procurement.

Individuals vaccinated through PhilHealth coverage received the vaccine (including its administration) free of charge. Only a single-dose vial presentation is currently available for PCV13, whereas PCV10 is available as a single dose and a two-dose vial presentation, with all presentations of both vaccines being prequalified by the WHO. The two dose preservative-free presentation of PCV10 is only available through UN procurement; the implementation of this vaccine presentation requires specific training for immunization staff as well as formal post-introduction monitoring [45]. For this analysis, it was assumed that a single-dose vial presentation for both vaccines would be procured. In terms of wastage, 5% of the total quantity of PCV10 and PCV13 was added according to WHO recommendations on vaccines with single dose presentation.

#### Other medical costs

Insurance claims data from 2012 were provided by PhilHealth and used to calculate the unit cost of hospitalized bacterial meningitis, hospitalized all-cause pneumonia, hospitalized all-cause sepsis and bacteremia (Table 1). WHO ICD 10 codes were used as reference to extract the total medical cost for each disease presentation.

Each insurance claim reflected both the actual total cost per case and the amount reimbursed by PhilHealth, excluding professional fees and other administration costs. For the base case analysis, the average of the actual total cost of all claims from public hospitals were considered, since PhilHealth reimbursement typically only covers a certain amount of the actual total cost per claim. Public hospital cost figures were adjusted by a 50% mark-up to account for administration cost, including professional fees in public hospitals (PhilHealth estimate). Private hospital cost significantly exceeded public hospital cost and were not regarded to be a reasonable proxy of actual medical cost. In sensitivity analysis, private hospital costs were taken into account by using a weighted average of public and private hospital costs.

Due to a lack of local studies and considering that the number of claims for relatively rare sequelaes due to *S*. *pneumoniae* may not be representative of the total number of cases in the country, the cost per year of these conditions were obtained from Thai cost data as presented in the economic evaluation of PCV 10 and 13 in Thai context [14]. 2010 Thai figures and 2012 PhilHealth figures were adjusted to 2013 for inflation and PPP (1 THB = 1.54 PHP), using the EPPI Centre Cost Converter as of 27 January 2014 [46].

#### Utilities

Due to absence of local utility parameters, Thai values using the Health Utilities Index Mark 3 were adopted [47].

### Analysis

One-way sensitivity analysis was performed to explore the robustness of the results to variations in uncertain key assumptions. The following alternative scenarios were assessed: discount rate at 0% and 10% per annum; duration of vaccine protection of 10 years; weighted average of public and private treatment cost based on PhilHealth claims; serotype replacement by using variation in vaccine serotype coverage; exclusion of herd protection; vaccine price using higher market prices; 40:60 ratio of hospitalized to non-hospitalized pneumonia cases; and exclusion of serotype 3 from local serotype coverage of PCV13 due to recent effectiveness data from the UK suggesting that PCV13 may be ineffective against serotype 3 [48].

Probabilistic sensitivity analysis was conducted using the Monte Carlo simulation using Microsoft Office Excel 2007. According to the feasible range of values each parameter could attain, the following probability distributions were used: the beta distribution was the choice of distribution when parameter values ranged between zero to one. The gamma distribution was used when parameter values ranged between zero to infinity, and the normal distribution was used when data were symmetrically distributed. A 1,000 iterations were run to yield possible values for costs, health outcomes and incremental cost-effectiveness ratios (ICERs). Acceptability curves were generated, showing the probability of each intervention being cost-effective at different ceiling threshold values.

A ceiling threshold of one per capita gross domestic product or Php 120,000 per QALY gained was used to determine the cost-effectiveness of each intervention as recommended by the FEC [49]. Budget impact analysis was performed to forecast the financial impact for a 5-year horizon of implementing PCV for either universal or partial coverage.

## Results

**Fig 3:**
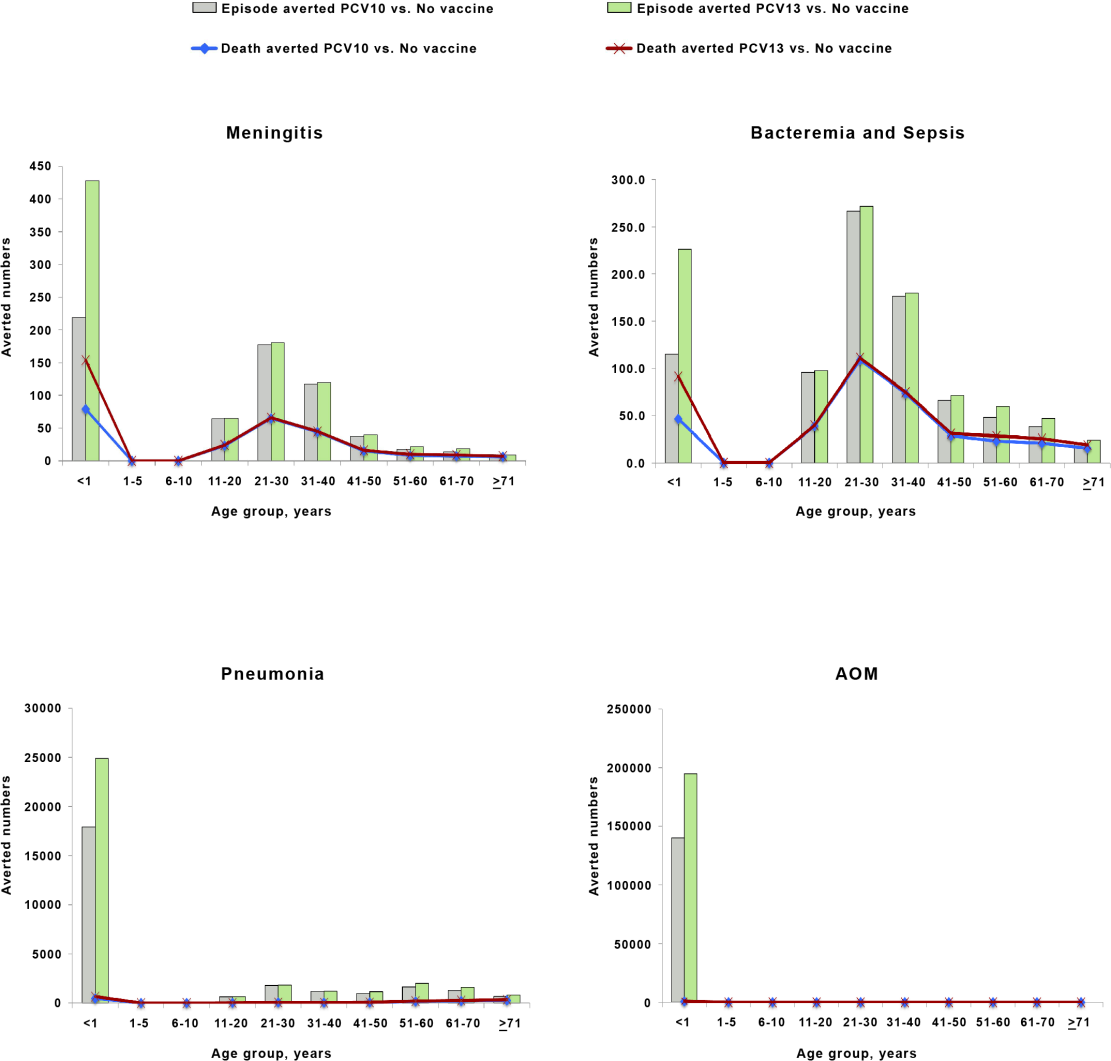
Predicted numbers of life-time pneumococcal disease cases and deaths averted due to vaccination with PCV10 and PCV13 by clinical syndrome and age at entry to the cohort.

Universal child vaccination with PCV would reduce both the clinical and the economic burden caused by *S*. *pneumoniae* infection. For an entire vaccinated birth cohort of 2 million infants, the 2+1 dose schedule of PCV10 and PCV13 was estimated to avert 334 and 654 episodes of IPD (meningitis, bacteremia) in the vaccinated population, respectively, compared to no vaccination (Fig 3). Indirect benefits of vaccination among the unvaccinated population, including the elderly, would prevent an additional 1,145 and 1,204 episodes of IPD (meningitis, bacteremia). Furthermore, using PCV10 and PCV13 would avoid 140,107 and 194,782 episodes of AOM, and 26,096 and 34,140 episodes of clinical pneumonia, respectively. In addition, it is estimated that implementing PCV10 and PCV13 would prevent 1,904 and 2,399 deaths, respectively.

Table 3 illustrates the ICERs of a 2+1 dose schedule of PCV10 and PCV13 for both scenarios. Considering the country-specific threshold of Php 120,000 per QALY gained, vaccinating the entire birth cohort with PCV10 and PCV13 (Scenario 1) would be highly cost-effective, producing ICERs of Php 68,182 and Php 54,510 per QALY gained, respectively, compared to no vaccination. Compared to PCV10, PCV13 generates better outcomes in terms of QALYs, life years gained and deaths averted with an ICER of Php 15,795.

**Table 3:** Incremental outcomes of introducing PCV10 and PCV13 compared to no vaccination.

	PCV 10 vs. no vaccination				PCV 13 vs. no vaccination				PCV 13 vs. PCV 10			
	Inc. Cost	Inc. LYs	Inc. QALYs	ICER/ QALY (Php)	Inc. Cost	Inc. LYs	Inc. QALYs	ICER/ QALY (Php)	Inc. Cost	Inc. LYs	Inc. QALYs	ICER/ QALY (Php)
**With herd protection** Scenario 1 (Universal coverage)	1491	0.01174	0.02186	**68,182**	1613	0.015222	0.029584	**54,510**	122	0.003482	0.007724	**15,795**
**Without herd protection** Scenario 2 (25/0)	440	0.001418	0.003906	**112,640**	483	0.00217	0.00571	**84,654**	43	0.000752	0.001804	**23,836**

LY- life years

QALY- Quality adjusted life year

In scenario 2, where indirect effects of vaccination were excluded due to partial vaccination coverage that is fully funded by the government, ICER values of PCV10 (Php 112,640) and PCV13 (Php 84,654) increased significantly, yet were still below the cost-effectiveness threshold of Php 120,000.

**Fig 4:**
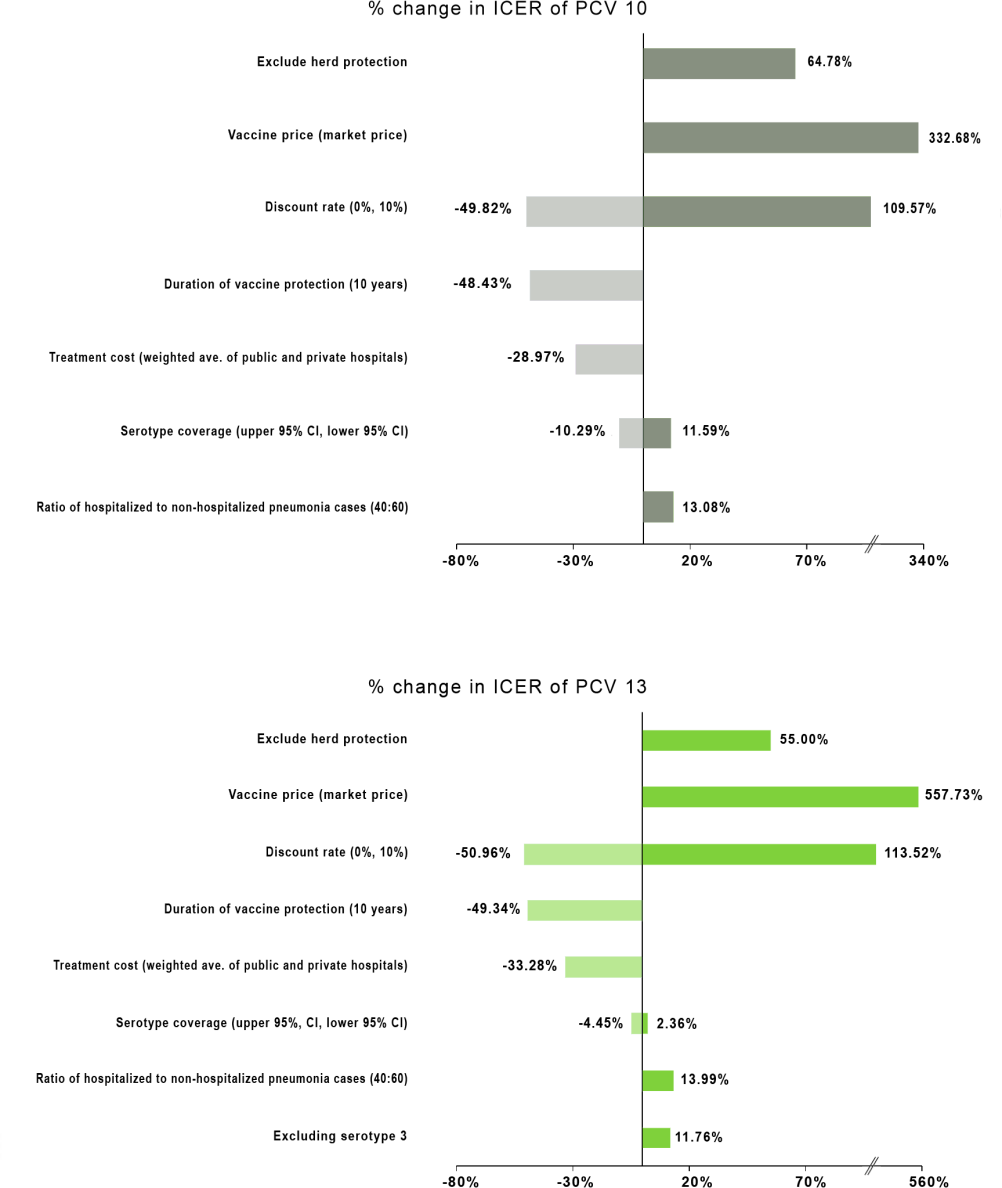
Tornado diagram for PCV10 and PCV13 versus no vaccination. Exploring the impact of uncertainty in key model parameters on ICERs of PCV10 (Php 68,182) and PCV13 (Php 54,510) in the scenario of universal coverage.

In one-way sensitivity analysis for scenario 1, selected parameters had a significant impact on ICERs, except serotype coverage, treatment cost (weighted average of public and private), and ratio of inpatient to outpatient pneumonia cases (40:60) (Fig 4). Substantial variations in ICER values were obtained with vaccine cost, exclusion of indirect effects, vaccine efficacy, and duration of vaccine protection. The discount rate may be considered as the single most significant parameter affecting the value for money of both vaccines. A small change in the discount rate resulted in a disproportionately larger change in the ICERs. Assuming there is no efficacy of PCV13 against serotype 3, the ICER of PCV13 increased by 11.76% to Php 60,921.

**Fig 5:**
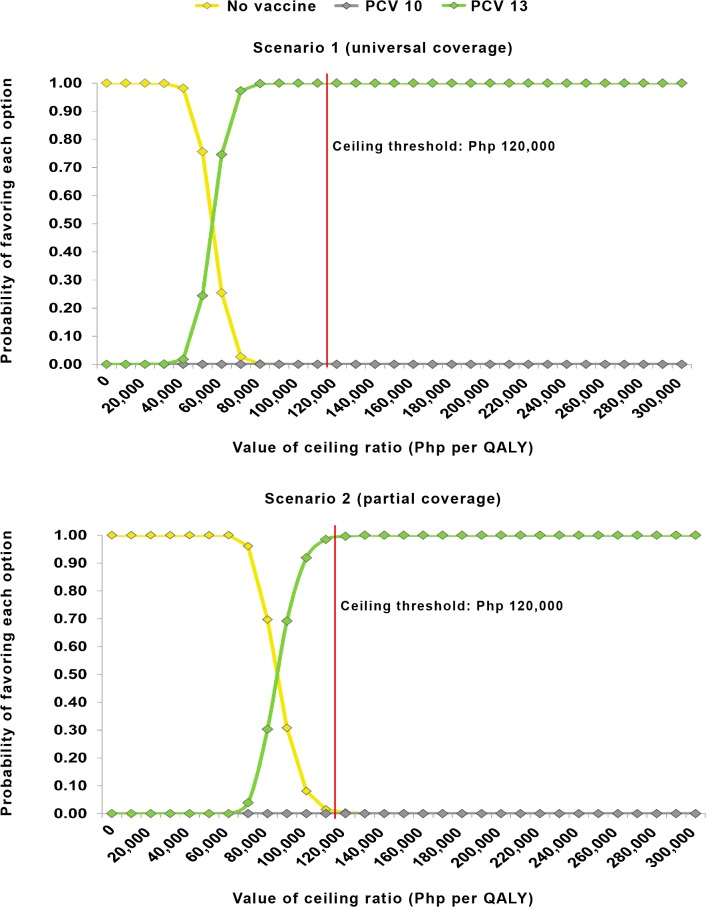
Cost-effectiveness acceptability curves for PCV10, PCV13 and no vaccination in the scenarios of universal and partial coverage.

Fig 5 demonstrates that adopting either universal or partial access to PCV (as compared to no vaccination) offers good value for money for the DOH, given the current ceiling threshold of Php 120,000 per QALY gained. For both scenarios, PCV13 yielded a 100% probability of being cost-effective compared to no vaccination and to PCV10 at the given ceiling threshold.

Threshold analysis revealed that in scenario 1, PCV10 and PCV13 prices would have to be 79% and 76% lower (to Php 131 and Php 166) in order to be cost-saving (ICER = 0) for the Philippines. In scenarios 2, the maximum vaccine costs for PCV10 and PCV13 would have to range from Php 42 to Php 60, respectively.

Table 4 presents the 5-year budget impact of implementing PCV10 and PCV13 for scenarios 1 and 2. Including PCV10 and PCV13 in the EPI for nationwide coverage requires an additional budget of Php 18.37 billion (US$ 416 million) and Php 20.41 billion (US$ 462 million), respectively, compared to no vaccination. Implementing PCV10 and PCV13 for only 25% of the birth cohort would require additional costs of Php 4.59 billion (US$ 104 million) and Php 5.11 billion (US$ 116 million), with fixed treatment costs of Php 30.98 billion (US$ 701 million) if no vaccination is introduced.

**Table 4:** Budget impact analysis of scenario 1 and 3 compared to no vaccination in billions (Php).

	Scenario 1 (Universal vaccination coverage)				Scenario 2 (25% vaccination coverage)				No Vaccination Program
	PCV 10		PCV 13		PCV 10		PCV 13		
	Vaccination cost	Treatment cost	Vaccination cost	Treatment cost	Vaccination cost	Treatment cost	Vaccination cost	Treatment cost	Treatment cost
**Year 1**	3.87	6.34	4.34	6.32	0.97	6.43	1.09	6.42	6.45
**Year 2**	3.87	5.87	4.34	5.83	0.97	5.99	1.09	5.97	6.02
**Year 3**	3.87	5.92	4.34	5.86	0.97	6.06	1.09	6.05	6.11
**Year 4**	3.87	5.93	4.34	5.84	0.97	6.10	1.09	6.08	6.17
**Year 5**	3.87	5.94	4.34	5.84	0.97	6.14	1.09	6.12	6.23
**5 year Budget Impact**	19.35	30.00	21.70	29.69	4.85	30.72	5.45	30.64	30.98
**Total Budget Impact**	49.35		51.39		35.57		36.09		30.98
**Incremental Budget**	18.37		20.41		4.59		5.11		

## Discussion

This study is the first economic evaluation conducted in the Philippines and to our best knowledge, it is the first in the world conducted after the release of the newest clinical evidence on efficacy of PCV10 published in 2014 [13]. It shows that at current vaccine pricing and ceiling threshold, introducing universal vaccination with either PCV10 or PCV13 would be cost-effective, compared to no vaccination. Introducing universal PCV vaccination throughout the country would cost Php 3.87–4.34 billion annually, or 1.6 to 1.8 times the budget currently allocated for the EPI. Hence, universal vaccination will only be feasible if additional budget for the EPI program is allocated, or vaccine prices are lowered. The current allocated budget of the NIP would suffice to vaccinate only 25% of the birth cohort.

Considering between the two vaccines, this study indicates that PCV13 achieves better value for money compared to PCV10, thus, PCV13 should be a better choice in the Philippines. The model was very sensitive to vaccine price and indirect effects (herd protection). However, while the use of current market prices for PCV10 and PCV13 made vaccination cost-ineffective, excluding herd protection generated ICER values that remained below the country-specific threshold. Excluding serotype 3 from the serotype coverage of PCV13 generated an ICER still below the base case ICER of PCV10. Apart from economic reasons, PCV13 is superior to PCV10 in terms of its broader coverage of serotypes; a universal vaccination program with PCV13 would lower the potential for serotype replacement. This phenomenon has been experienced in many countries including the USA and UK, where a dramatic increase in IPD caused by non-vaccine serotypes following PCV7 introduction offset some of the benefits of vaccination [5, 50]. Serotype replacement was particularly observed for the multi-resistant serotype 19A [51–53], which is only covered by PCV13. As a consequence, many Western countries replaced the earlier version of PCV7 with PCV13 in their national immunization programs due to the rapid rise of this virulent serotype in invasive pneumococcal isolates [54]. Serotype 19A was also one of the more commonly isolated serotypes in Filipino children under 5 years of age [27]. A case-control study of PCV7 effectiveness and studies comparing immunogenicity of PCV10 and PCV7 suggest a potential cross-reactivity effect of PCV10 against serotypes 6A and 19A, due to the contained antigens in PCV10 against serotype 6B and 19F [55, 56]. However, cross-reactivity results of PCV10 based on the COMPAS were not significant (cross-reactive serotypes IPD [6A, 9N, or 19A] -99.5% {95% CI: -2,100.2–81.9} and AOM [6A, 18B, 19A, or 23A] 29% {95% CI: -123.7–77.5}) [13], cross-protection was therefore not considered in the model.

This cost-effectiveness study used a similar analytic approach to that used in Thailand [14], in terms of the static model structure and utility values considered, and methods used to derive vaccine efficacy and indirect effects. However, in contrast to the Philippines’ findings, Thai results showed that neither PCV10 nor PCV13 were cost-effective at the ceiling threshold of THB 100,000 (US$ 3,226). The differences may be explained by differences in vaccine price, epidemiological parameters, and vaccine efficacy used in the model. Thai government vaccine prices per dose were significantly higher (THB 1,440 or US$ 46 for PCV10 and THB 1,930 or US$ 62 for PCV13), whereas Thai IPD incidence, mortality rates, and AOM efficacy values were lower. Results of this study are similar to previous studies conducted in Taiwan and Singapore, which found PCV13 to be cost-effective [9, 11, 42]. In Singapore, pneumococcal conjugate vaccination was a cost-effective intervention only if herd protection effects were considered, resulting in ICER values comparable to the country’s GDP per capita. The cost per QALY of PCV13 was lower compared to PCV10; however, if PCV10 was attributed a protective effect against NTHi AOM, it was found to be more cost-effective compared to PCV13.

This study has some limitations. First, due to lacking clinical trial data against final clinical endpoints, vaccine efficacy for PCV13 was extrapolated from PCV10 efficacy trials, which may under- or overestimate its efficacy. Second, this study did not employ dynamic modeling, which is generally recommended by cost-effectiveness analysis guidelines in order to account for externalities. However, this study used an excel-based static model (Markov), which accounted for indirect effects of vaccination. The use of static model facilitated transparency in terms of the methods used in this study because many local decision makers are familiar with this type of modeling. Third, since no local data on indirect vaccine effects were available, US data were used and adjusted to local serotype coverage. However, US findings showed a statistically significant decline in IPD incidence among unvaccinated persons aged 20 years and above only. Thus, our study did not include indirect effects among populations under 20 years. Fourth, the only available study on IPD incidence in the country was hospital-based [16], with incidences weighted by the number of children in the at-risk population, which may have led to an overestimation of IPD incidence rates used in the model. On the contrary, lower bacterial isolation rates attributed to high rates of antimicrobial use by parents of young children in the Philippines may have led to an underestimation of IPD [16]. Fifth, results of this study are dependent on the local serotype distribution and on the country-specific ceiling threshold, with the latter being based on the preferences of decision makers in the Philippines. Therefore, applying results of this study to other settings or populations should be performed with caution. Sixth, we assumed all AOM cases are treated on an outpatient basis, resulting in an underestimation of the cost of AOM treatment because in some severe cases, hospitalization for tube replacement may be required. However, including hospitalization cost for AOM will not change our overall conclusion as higher treatment cost will result in a lowering of the ICERs, which will further favor vaccination. Lastly, differences in pathogenicity between pneumococcal serotypes are important when evaluating the benefits of pneumococcal conjugate vaccines of different valency [57]. This model did not account for differences between serotypes in terms of their propensity to cause morbidity or death.

## Supporting Information

S1 Table Philippine vaccine-type IPD coverage of PCV7, PCV10, and PCV13 in different age groups. (DOCX)

S2 Table Summary of % reduction in IPD and Pneumonia incidences in the Philippines (PHL) based on US data. (DOCX)
