# Supplementary material for: Do Pneumococcal Conjugate Vaccines Represent Good Value for Money in a Lower-Middle Income Country? A Cost-Utility Analysis in the Philippines
Source: PLoS One. 2015 Jul 1;10(7):e0131156. doi: 10.1371/journal.pone.0131156 (PMC4488861; doi:10.1371/journal.pone.0131156)
Supplement: S1 Table — (DOCX) [file pone.0131156.s001.docx]

| **PHILIPPINE VACCINE SEROTYPE COVERAGE** | | | | | | | | | | | | | | | |
| --- | --- | --- | --- | --- | --- | --- | --- | --- | --- | --- | --- | --- | --- | --- | --- |
| **SEROTYPE** | **< 5 (n= 93)** | | | **6 to 18 (n=105)** | | | **19 to 49 (n=71)** | | | **> 50 (n=32)** | | | **TOTAL (N=301)** | | |
|  | PCV 7 | PCV 10 | PCV 13 | PCV 7 | PCV 10 | PCV 13 | PCV 7 | PCV 10 | PCV 13 | PCV 7 | PCV 10 | PCV 13 | PCV 7 | PCV 10 | PCV 13 |
| **4** | 4.30% | 4.30% | 4.30% | 3.13% | 3.13% | 3.13% | 4.23% | 4.23% | 4.23% | 9.52% | 9.52% | 9.52% | 5.98% | 5.98% | 5.98% |
| **6B** | 9.68% | 9.68% | 9.68% | 6.25% | 6.25% | 6.25% | 0.00% | 0.00% | 0.00% | 2.86% | 2.86% | 2.86% | 4.65% | 4.65% | 4.65% |
| **9V** | 0.00% | 0.00% | 0.00% | 0.00% | 0.00% | 0.00% | 1.41% | 1.41% | 1.41% | 0.95% | 0.95% | 0.95% | 0.66% | 0.66% | 0.66% |
| **14** | 11.83% | 11.83% | 11.83% | 3.13% | 3.13% | 3.13% | 7.04% | 7.04% | 7.04% | 0.95% | 0.95% | 0.95% | 5.98% | 5.98% | 5.98% |
| **18C** | 1.08% | 1.08% | 1.08% | 3.13% | 3.13% | 3.13% | 0.00% | 0.00% | 0.00% | 0.95% | 0.95% | 0.95% | 1.00% | 1.00% | 1.00% |
| **19F** | 1.08% | 1.08% | 1.08% | 3.13% | 3.13% | 3.13% | 0.00% | 0.00% | 0.00% | 2.86% | 2.86% | 2.86% | 2.99% | 2.99% | 2.99% |
| **23F** | 6.45% | 6.45% | 6.45% | 6.25% | 6.25% | 6.25% | 2.82% | 2.82% | 2.82% | 1.90% | 1.90% | 1.90% | 3.99% | 3.99% | 3.99% |
| **1** |  | 6.45% | 6.45% |  | 18.75% | 18.75% |  | 30.99% | 30.99% |  | 14.29% | 14.29% |  | 16.28% | 16.28% |
| **5** |  | 9.68% | 9.68% |  | 25.00% | 25.00% |  | 22.54% | 22.54% |  | 9.52% | 9.52% |  | 14.29% | 14.29% |
| **7F** |  | 0.00% | 0.00% |  | 0.00% | 0.00% |  | 2.82% | 2.82% |  | 5.71% | 5.71% |  | 2.66% | 2.66% |
| **3** |  |  | 5.38% |  |  | 3.13% |  |  | 0.00% |  |  | 6.67% |  |  | 4.32% |
| **6A** |  |  | 6.45% |  |  | 3.13% |  |  | 1.41% |  |  | 2.86% |  |  | 3.65% |
| **19A** |  |  | 7.53% |  |  | 0.00% |  |  | 0.00% |  |  | 1.90% |  |  | 2.99% |
| GRAND TOTAL | 34.41% | 50.54% | 69.89% | 25.00% | 68.75% | 75.00% | 15.49% | 71.83% | 73.24% | 20.00% | 49.52% | 60.95% | 25.25% | 58.47% | 69.44% |

**Supplementary Table 1. Philippine vaccine-type IPD coverage of PCV7, PCV10, and PCV13 in different age groups**
