# Supplementary material for: Do Pneumococcal Conjugate Vaccines Represent Good Value for Money in a Lower-Middle Income Country? A Cost-Utility Analysis in the Philippines
Source: PLoS One. 2015 Jul 1;10(7):e0131156. doi: 10.1371/journal.pone.0131156 (PMC4488861; doi:10.1371/journal.pone.0131156)
Supplement: S2 Table — (DOCX) [file pone.0131156.s002.docx]

**Supplementary Table 2. Summary of % reduction in IPD and Pneumonia incidences in the Philippines (PHL) based on US data.**

| **AGE** | **PCV10** | | **PCV13** | |
| --- | --- | --- | --- | --- |
|  | **IPD^a^** | **Pneumonia^b^** | **IPD^a^** | **Pneumonia^b^** |
| 0 | 0.00% | 0.00% | 0.00% | 0.00% |
| 1 | 0.00% | 0.00% | 0.00% | 0.00% |
| 6 | 0.00% | 0.00% | 0.00% | 0.00% |
| 20 | 40.30% | 2.84% | 41.09% | 2.90% |
| 40 | 15.38% | 1.09% | 15.68% | 1.11% |
| 50 | 10.60% | 0.75% | 13.05% | 0.92% |
| 65 | 20.61% | 1.45% | 25.36% | 1.79% |

1. $\% IPD fall in PHL=\% IPD fall in US\times\frac{serotype coverage in PHL}{serotype coverage in US}$
2. $\% Hospitalized pneumonia fall in PHL=Proportion of pneumococcal pneumonia\times\% IPD fall in PHL$
